# *Bacillus amyloliquefaciens* FH-1 significantly affects cucumber seedlings and the rhizosphere bacterial community but not soil

**DOI:** 10.1038/s41598-021-91399-6

**Published:** 2021-06-08

**Authors:** Jingjing Wang, Song Xu, Rong Yang, Wei Zhao, Dan Zhu, Xiaoxia Zhang, Zhiyong Huang

**Affiliations:** 1 Tianjin Key Laboratory for Industrial Biological Systems and Bioprocessing Engineering, Tianjin Institute of Industrial Biotechnology, Chinese Academy of Sciences, Tianjin, 300308 People’s Republic of China; 2 National Technology Innovation Center of Synthetic Biology, Tianjin, 300308 People’s Republic of China; 3 Tianjin Institute of Industrial Biotechnology, Chinese Academy of Sciences, No. 32, West 7th Avenue, Tianjin Airport Economic Area, Tianjin, 30030 People’s Republic of China

**Keywords:** Microbial ecology, Soil microbiology

## Abstract

Plant growth-promoting bacteria (PGPB) inoculants have been applied worldwide. However, the ecological roles of PGPB under different soil conditions are still not well understood. The present study aimed to explore the ecological roles of *Bacillus amyloliquefaciens* FH-1 (FH) on cucumber seedlings, rhizosphere soil properties, and the bacterial community in pot experiments. The results showed that FH had significant effects on cucumber seedlings and the rhizosphere bacterial community but not on soil properties. The FH promoted cucumber seedlings growth, reduced the rhizosphere bacterial diversity, increased Proteobacteria, and decreased Acidobacteria. Linear discriminant analysis (LDA) effect size (LEfSe) revealed that FH enriched two taxa (GKS2_174 and Nannocystaceae) and inhibited 18 taxa (mainly Acidobacteria, Actinobacteria, BRC1, Chloroflexi, Plantctomycetes, and Verrucomicrobia). Co-occurrence network analysis demonstrated that FH increased bacteria-bacteria interactions and that *Bacillus* (genus of FH) had few interactions with the enriched and inhibited taxa. This might indicate that FH does not directly affect the enriched and inhibited taxa. Correlation analysis results displayed that cucumber seedlings’ weight and height/length (except root length) were significantly correlated with the 18 inhibited taxa and the enriched taxa Nannocystaceae. It was speculated that FH might promote cucumber seedling growth by indirectly enriching Nannocystaceae and inhibiting some taxa from Acidobacteria, Actinobacteria, BRC1, Chloroflexi, Plantctomycetes, and Verrucomicrobia.

## Introduction

Cucumber is an important vegetable in many countries, including China. Due to the higher requirements, higher productivity of cucumbers relies heavily on chemical fertilizers and pesticides^1^. With increasing pollution and costs of chemical fertilizers and pesticides, plant growth-promoting bacteria (PGPB) inoculants are advantageous for the development of sustainable agriculture^2,3^. A substantial number of PGPB inoculants have been applied and commercialized for various crops worldwide^4–6^. PGPB mainly promote the growth of plants by providing nutrients, secreting hormones, antagonizing pathogens, and resisting stress^7,8^. However, poor productivity and stability impede the large-scale application of microbial inoculants in mainstream agriculture^9,10^. Understanding the ecological roles of the PGPB in the complex soil system may guide the development and application of PGPB inoculants in future.

*Bacillus amyloliquefaciens* is known for its ability to suppress plant pathogens and promote plant growth^11,12^. It has been widely applied on rice, tomato, cucumber, and lettuce, among others^13–15^. Many studies have demonstrated that *B*. *amyloliquefaciens* can reduce the incidence or severity of various diseases on a diversity of hosts^13,16,17^. This might be related to the secretion of antimicrobial lipopeptides, antibiotics, and hydrolases and might also be related to the regulation of the rhizosphere microbiome^12,13^. Many reports have shown that *B. amyloliquefaciens* can promote the growth of crops and improve the yield and quality of crops. This might be related to the secretion of indoleacetic acid (IAA), the improvement of available nutrients in soil through nitrogen fixation, phosphorus removal, and potassium dissolving, and the regulation of the rhizosphere microbiome^18–20^.

In recent years, with the recognition of the importance of the rhizosphere microbiome, research on the effect of *B. amyloliquefaciens* on the rhizosphere microbiome has increased. Rhizosphere microbiomes play key roles in the disease, health, growth, and development of their host^21–24^. Many reports have indicated that the application of microbial inoculants could influence resident microbial communities^8,25–27^. The effects of *B. amyloliquefaciens* on the rhizosphere microbial communities of tomato, rice, lettuce, banana, tobacco, and cucumber were investigated (Table S3). However, most studies focus on community composition and diversity, while only a few focus on co-occurrence network analysis. Co-occurrence network analysis of taxon co-occurrence patterns might help identify potential biotic interactions between inoculants and soil indigenous microorganisms and increase the understanding of how inoculants affect microbial communities^13,28,29^. In addition, many studies on cucumbers are based on peat and vermiculite^13,19^. This may be different from the results based on soil. Moreover, the ecological roles of *B. amyloliquefaciens* under soil conditions are not well understood. The comprehensive effects of *B. amyloliquefaciens* on crops, soil, and microorganisms still lack systematic and in-depth study.

To better understand the ecological roles of *B. amyloliquefaciens* under soil conditions, we investigated the effects of *B. amyloliquefaciens* FH-1 (FH), which could significantly promote rice growth in field experiments^18^, on cucumber seedlings, rhizosphere soil properties, and the bacterial community in soil by using high-throughput sequencing technology, network analysis, and multivariate statistical methods. This will provide theoretical guidance for the development and application of PGPB inoculants in future.

## Results

### FH had significant effects on cucumber seedlings

The cucumber seedlings’ weight and height were significantly affected by FH (Table 1). FH significantly increased the fresh weight of plants, shoots, and roots and increased the plant dry weight and shoot height of cucumber seedlings compared to those drenched with sterile deionized water (CK).

**Table 1:** Effects of *Bacillus amyloliquefaciens* FH-1 inoculation on cucumber seedlings.

Cucumber seedlings	CK	FH
**Fresh weight (g)**		
*Plant*	1.90 ± 0.20b	3.25 ± 1.04a
*Shoot*	1.71 ± 0.19b	2.82 ± 0.90a
*Root*	0.19 ± 0.04b	0.43 ± 0.17a
**Dry weight (g)**		
*Plant*	0.15 ± 0.06b	0.37 ± 0.17a
Shoot	0.12 ± 0.06a	0.25 ± 0.11a
Root	0.03 ± 0.01a	0.12 ± 0.09a
**Height/length (cm)**		
Plant	14.22 ± 1.09a	16.23 ± 2.00a
*Shoot*	9.73 ± 0.33b	11.65 ± 1.55a
Root	4.49 ± 1.00a	4.58 ± 0.65a

Values (means ± SD, n = 5) within the same row followed by different letters are significantly different at *P* < 0.05 according to Independent-Samples t Test.

*CK* non-inoculated, *FH* inoculated with *Bacillus amyloliquefaciens* FH-1.

### FH had no significant effect on rhizosphere soil properties

FH had no significant effect on soil pH, total organic carbon, total nitrogen, total phosphorus, nitrate nitrogen, or available phosphorus (Table 2). However, the soil total nitrogen, total phosphorus, nitrate nitrogen, and available phosphorus in FH were generally higher than that in CK.

**Table 2:** Effects of *Bacillus amyloliquefaciens* FH-1 inoculation on rhizosphere soil properties.

	CK	FH
pH	8.48 ± 0.07a	8.46 ± 0.09a
TOC (g/kg)	6.44 ± 3.40a	4.08 ± 0.61a
TN (mg/kg)	733.20 ± 199.73a	765.60 ± 151.03a
TP (mg/kg)	311.86 ± 20.96a	342.35 ± 66.18a
NO_3_-N (mg/kg)	107.07 ± 16.23a	117.51 ± 20.82a
AP (mg/kg)	88.11 ± 0.95a	89.65 ± 1.62a

Values (means ± SD, n = 5) within the same row followed by different letters are significantly different at *P* < 0.05 according to Independent-Samples t Test.

*TOC* total organic carbon, *TN* total nitrogen, *TP* total phosphate, *NO*_*3*_*-N* nitrate nitrogen, *AP* available phosphate, *CK* non-inoculated, *FH* inoculated with *Bacillus amyloliquefaciens* FH-1.

### FH significantly affects rhizosphere bacterial community composition

**Figure 1:**
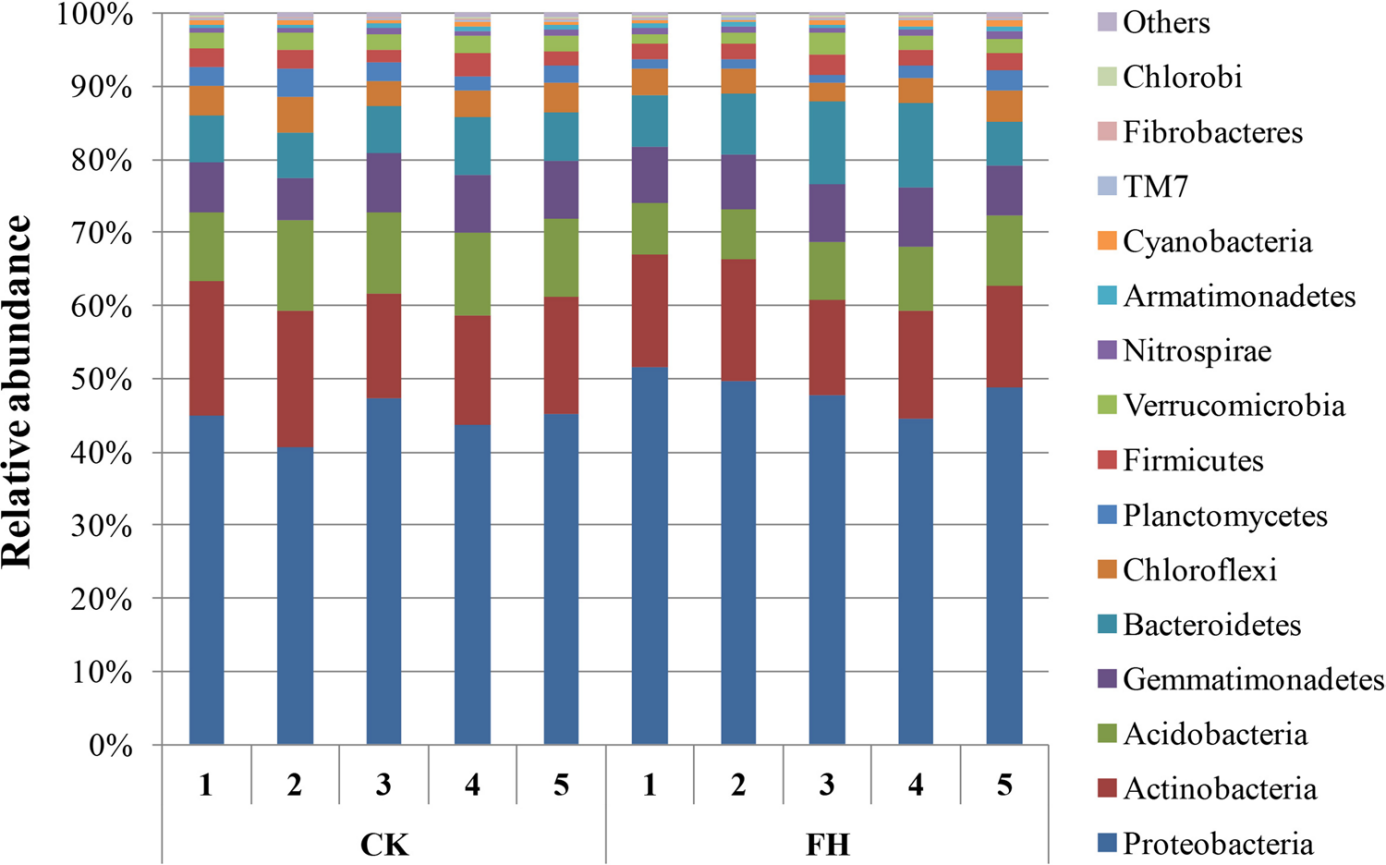
Relative abundance of the dominant rhizosphere bacterial phyla (proteobacterial classes) under different treatments. *CK* non-inoculated, *FH* inoculated with *Bacillus amyloliquefaciens* FH-1.

Across all samples, a total of 634,513 high-quality sequences and 57,039–68,492 sequences per sample (mean = 63,451) were obtained. After being rarefied to 57,000 sequences per sample, Alphaproteobacteria, Actinobacteria, Acidobacteria, Betaproteobacteria, Gammaproteobacteria, Deltaproteobacteria, Gemmatimonadetes, Bacteroidetes, Chloroflexi, Planctomycetes, Firmicutes, Verrucomicrobia, Nitrospirae, Armatimonadetes, Cyanobacteria, TM7, Fibrobacteres, and Chlorobi were found to be the dominant phyla (> 1%) across all treatments (Fig. 1). These dominant phyla accounted for more than 94% of the bacterial sequences from each soil sample. Deltaproteobacteria (*P* = 0.01) was significantly increased, while Acidobacteria (*P* = 0.00) was significantly decreased by FH (Table S1).

**Figure 2:**
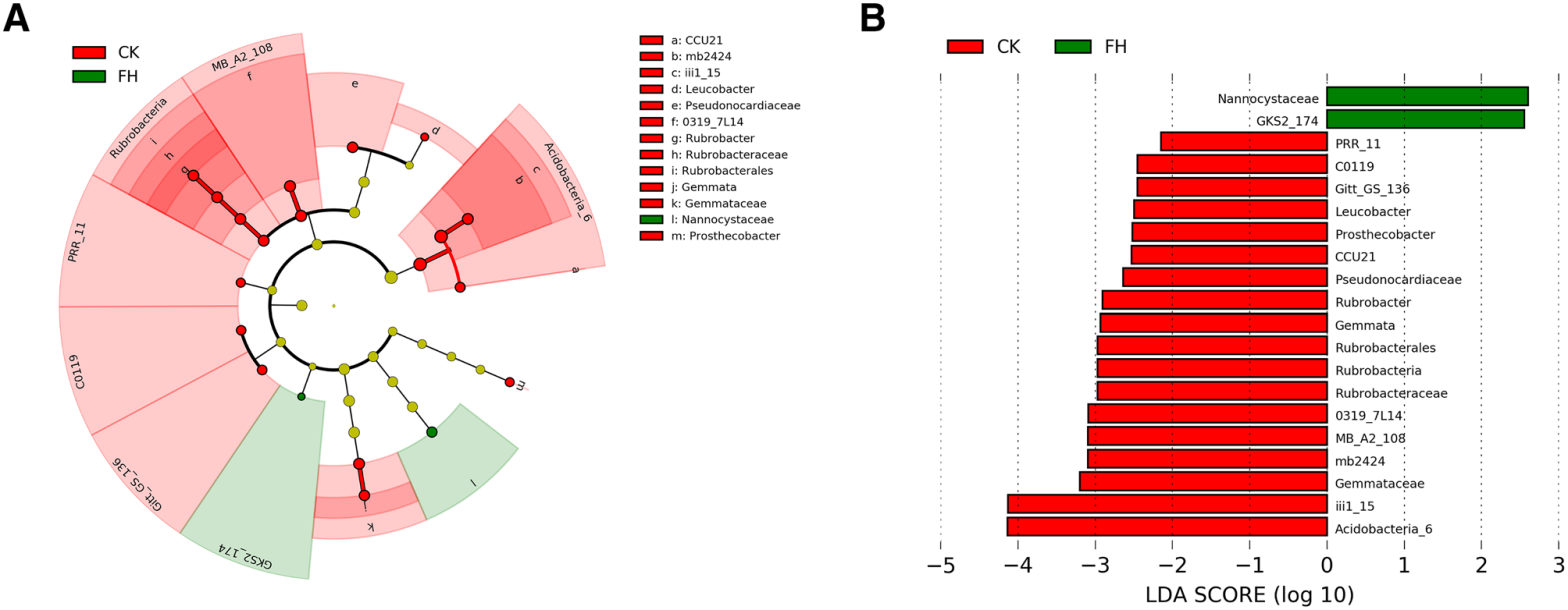
Cladogram (**A**) and linear discriminant analysis (LDA) score (**B**) of LEfSe analysis of the rhizosphere bacterial community between CK (red) and FH (green) treatments. *CK* non-inoculated, *FH* inoculated with *Bacillus amyloliquefaciens* FH-1.

LEfSe analysis showed that a total of 20 bacterial groups were distinct between FH and CK treatments using the logarithmic (LDA) value of 2 (Fig. 2). The bacterial taxa enriched in FH were GKS2-174 and Nannocystaceae. Acidobacteria-6 (the class and its order CCU21 and iii1-15, the order and its family mb2424), MB-A2-108 (the class and its order 0319-7L14), Rubrobacteria (the class and its order Rubrobacterales, the order and its family Rubrobacteraceae), PRR-11, C0119, Gitt-GS-136, Gemmataceae (the family and its genus *Gemmata*), Pseudonocardiaceae, *Leucobacter,* and Prosthecobacter were enriched in CK, which also could be regarded as inhibited taxa in FH.

The relative abundances of both *B. amyloliquefaciens* and *Bacillus* spp. were slightly higher in FH than in CK (Fig. S1). This suggested that *B. amyloliquefaciens* FH-1 might slightly colonize cucumber rhizosphere soil.

### FH had negative effects on rhizosphere bacterial diversity

The rhizosphere bacterial α-diversity was negatively affected by FH (Table 3). FH significantly decreased Observed_otus (*P* = 0.01) and PD_whole_tree (*P* = 0.02). Chao1 (*P* = 0.07) and the Shannon index (*P* = 0.10) were lower in FH than in CK.

**Table 3:** Effects of *Bacillus amyloliquefaciens* FH-1 inoculation on rhizosphere bacterial alpha diversity.

	CK	FH
Chao1	7176.53 ± 117.44a	6971.29 ± 159.56a
Observed_otus	4789.86 ± 98.59a	4599.68 ± 64.80b
PD_whole_tree	253.21 ± 3.46a	246.76 ± 2.38b
Shannon index	9.74 ± 0.11a	9.63 ± 0.07a

Values (means ± SD, n = 5) within the same row followed by different letters are significantly different at *P* < 0.05 according to Independent-Samples t Test.

*Chao1* richness of the Chao1 estimator, *Observed_otus* observed operational taxonomic units, *Shannon index* nonparametric Shannon diversity index, *CK* non-inoculated, *FH* inoculated with *Bacillus amyloliquefaciens* FH-1.

**Figure 3:**
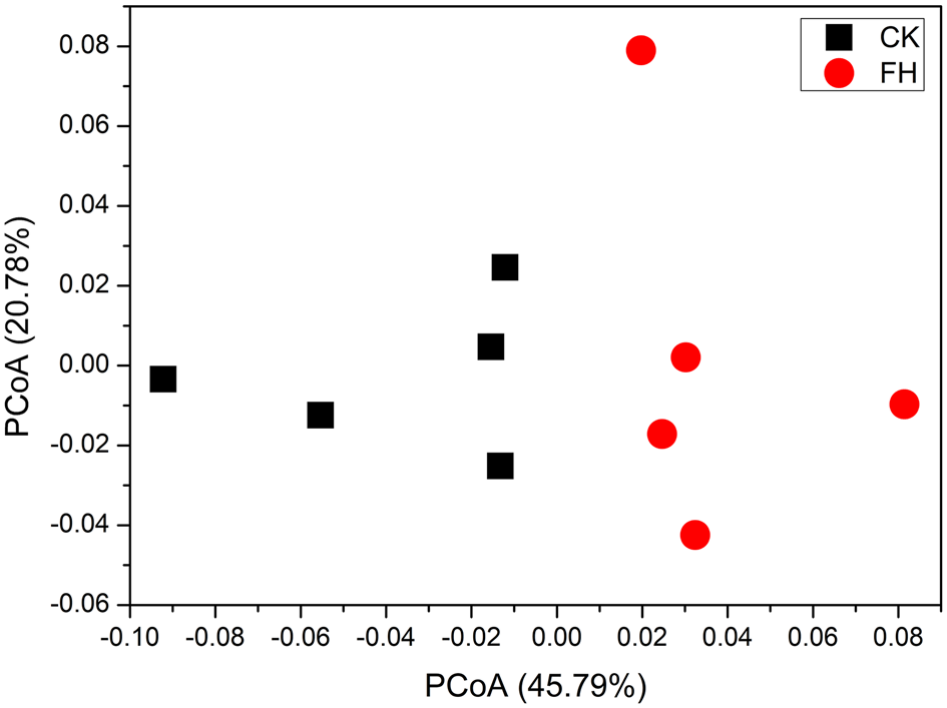
Principal coordinate analysis (PCoA) of weighted UniFrac distances of the rhizosphere bacterial community under different treatments. *CK* non-inoculated, *FH* inoculated with *Bacillus amyloliquefaciens* FH-1.

Principal coordinate analysis (PCoA) revealed that the rhizosphere bacterial communities of FH were distinct from those of CK (Fig. 3). ANOSIM analysis (global R = 0.488, *P* = 0.008) and PERMANOVA analysis (R^2^ = 0.326, *P* = 0.009) demonstrated that the structure of bacterial communities was significantly changed by FH.

### FH modified rhizosphere bacterial networks

**Figure 4:**
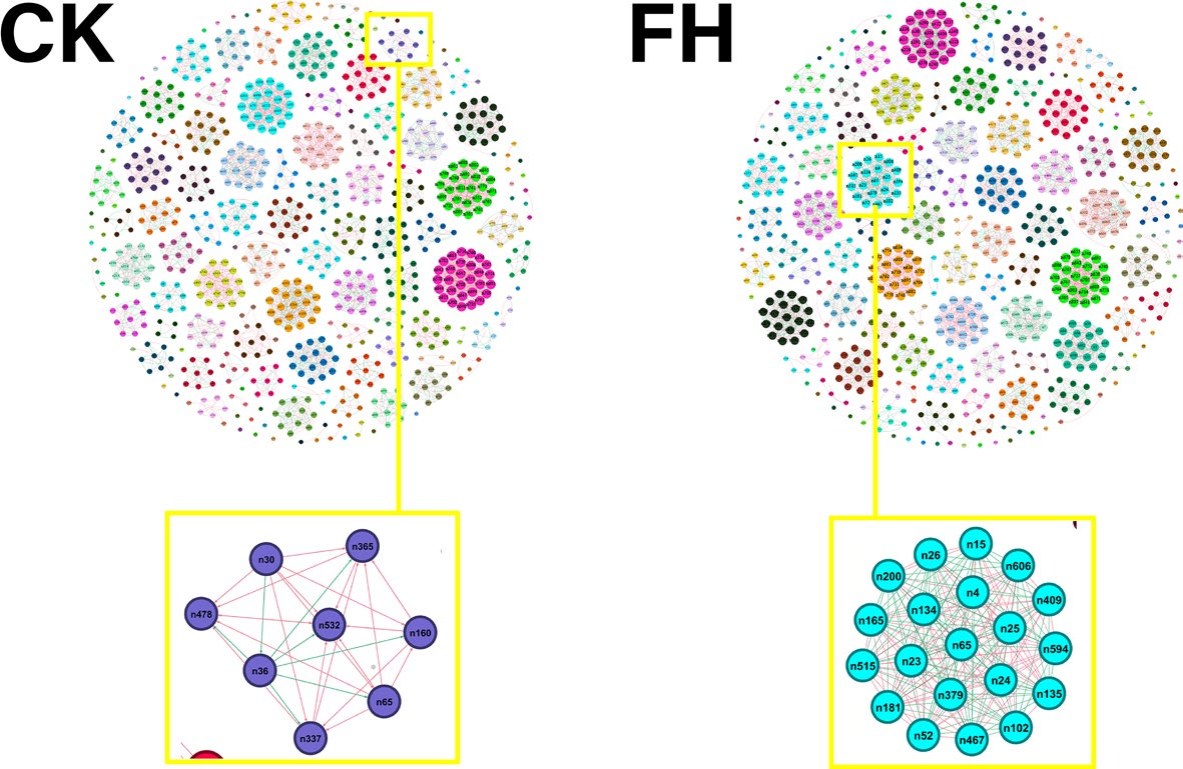
Networks of co-occurring rhizosphere bacterial genera in non-inoculated (CK) and *Bacillus amyloliquefaciens* FH-1 inoculated (FH) soil based on correlation analysis. A connection stands for a strong (Spearman's r > 0.6) and significant (*P* < 0.05) correlation. A blue edge indicates a negative interaction between two individual nodes, while a red edge indicates a positive interaction. The thickness of each connection between two nodes (i.e., edge) is proportional to the value of Spearman's correlation coefficient. The co-occurring networks are colored by modularity class. The size of each node is proportional to the number of connections (i.e., degree). *Bacillus* is labeled n65.

Whether FH affected the interaction of bacterial communities and whether FH interacted with enriched or inhibited taxa at the genus level were determined using co-occurrence network analysis based on a strong (Spearman's r > 0.6) and significant (*P* < 0.05) correlation. The calculated modularity index was larger than 0.4, and the random modularity index (Table 4) indicated a typical module structure^30^. Overall, the FH showed a remarkable influence on the co-occurrence networks in bacterial communities (Fig. 4). The number of positive correlations was higher than that of the negative correlations in both networks. FH had higher edges, negative correlations, and an average degree and modularity but lower positive correlations than CK (Table 4). There were more species interacting with *Bacillus* in FH than that in CK. There were 19 genera that interacted with *Bacillus*, and seven of them had positive interactions in FH. In CK, only seven genera interacted with *Bacillus*, and six of them had positive interactions (Fig. 4 and Table S2). *Bacillus* only had positive interactions with the inhibited taxa *Leucobacter* in CK and the inhibited taxa MB-A2-108 in FH.

**Table 4:** Topological properties of rhizosphere bacterial networks obtained from different treatments.

	CK	FH
**Empirical networks**		
Number of nodes	817	817
Number of edges	3963	4107
Number of positive correlations	2743 (69.22%)	2635 (64.16%)
Number of negative correlations	1220 (30.78%)	1472 (35.84%)
Average degree	4.851	10.054
Average clustering coefficient	1	1
Average path length	1	1
Network diameter	1	1
Graph density	0.012	0.012
Modularity	0.967	0.970
**Random networks**		
Average clustering coefficient	0.012 ± 0.001	0.012 ± 0.001
Average path length	3.202 ± 0.003	3.157 ± 0.002
Modularity	0.284 ± 0.004	0.278 ± 0.004

*CK* non-inoculated, *FH* inoculated with *Bacillus amyloliquefaciens* FH-1.

### Cucumber seedling characteristics were significantly correlated with the bacteria inhibited and enriched by FH

**Figure 5:**
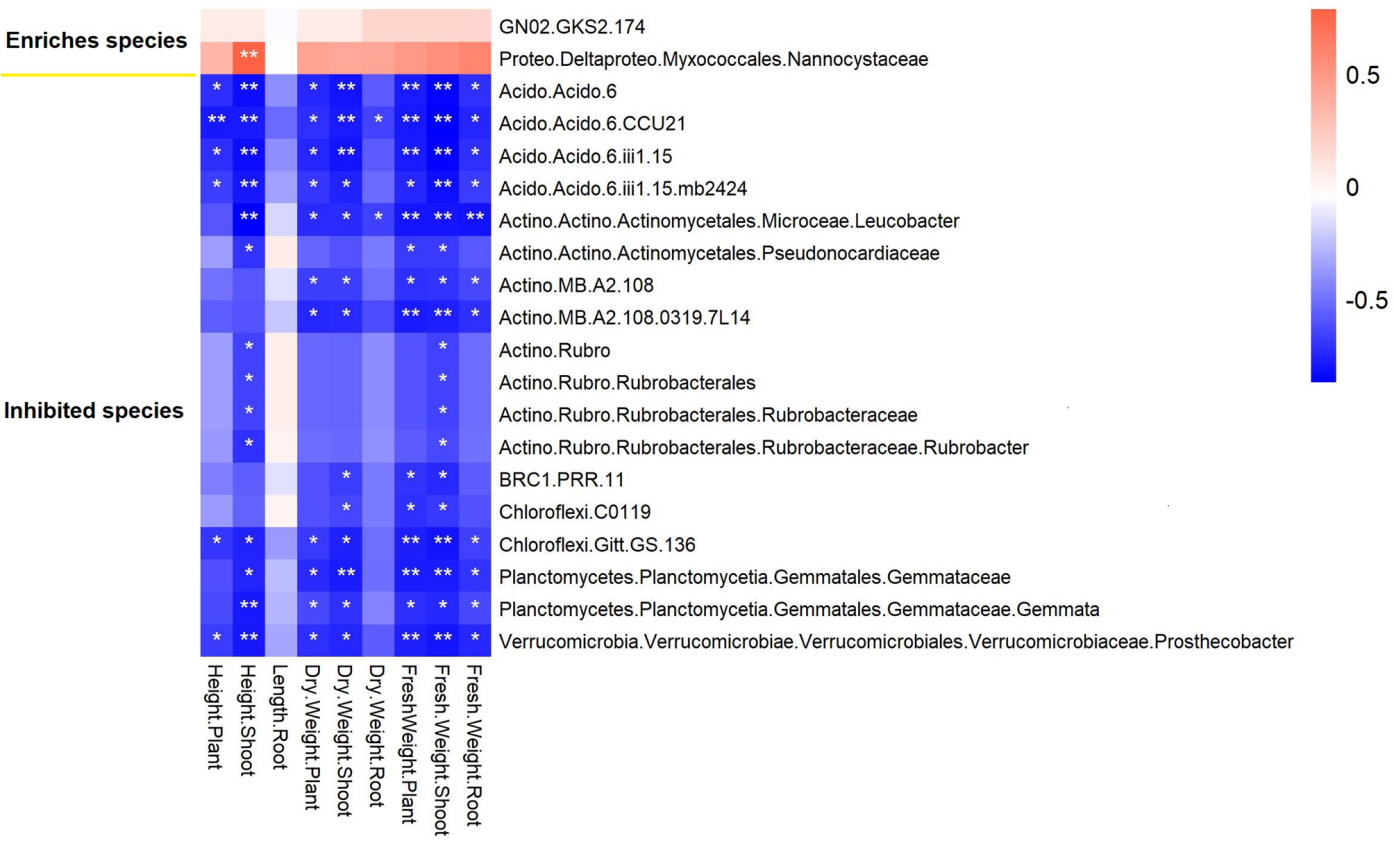
Heatmap of Spearman’s correlation coefficients between cucumber seedlings and bacteria inhibited and enriched by FH. The colors represent the correlation, with red being more positive and blue being more negative. Significance is given as *(*P* < 0.05) and **(*P* < 0.01).

Correlation analysis showed that cucumber seedlings’ weight and height/length (except root length) had a significant correlation with the bacteria taxa inhibited and enriched by FH (Fig. 5). All 18 inhibited taxa (mainly Acidobacteria, Actinobacteria, BRC1, Chloroflexi, Plantctomycetes, and Verrucomicrobia) were significantly and negatively correlated with some cucumber seedlings’ characteristics. These inhibited taxa had a closer relationship with cucumber shoots than roots. Enriched taxa Nannocystaceae had a significant positive correlation with cucumber shoot height.

## Discussion

In this study, the ecological roles of inoculant *B. amyloliquefaciens* FH-1 on cucumber seedlings, rhizosphere soil, and the bacterial community were investigated. The results illustrated that FH had a significant effect on cucumber seedlings and the rhizosphere bacterial community but not on soil.

Rhizosphere bacterial communities play a key role in the disease, health, growth, and development of plants^31–33^. The effect of PGPB on the bacterial community is still unclear. As a well-known PGPB, the effect of *B. amyloliquefaciens* on the bacterial community has been widely studied (Table S3). Some studies have shown that *B. amyloliquefaciens* has no influence on rhizosphere bacteria, while some have a significant influence. Some increased diversity, while some decreased diversity, and some improved Proteobacteria, while some improved Firmicutes^13–19,34–38^. In this study, we found that FH inoculation significantly reduced bacterial diversity, increased Proteobacteria that may belong to r-strategies, and decreased Acidobacteria that may belong to k-strategies^14^. The influence of *B. amyloliquefaciens* on the bacterial community may be attributed to different strains, plant species, soil types, and environmental factors. Therefore, it is necessary to investigate the influence of *B. amyloliquefaciens* on the bacterial community of the same crop in different soils or different crops in the same soil to reveal the regulation of *B. amyloliquefaciens* on the bacterial community.

LEfSe was used to identify taxa that were inhibited and enriched by FH. The inhibited taxa were mainly Acidobacteria, Actinobacteria, Chloroflexi, BRC1, Planctomycetes, and Verrucomicrobia. The inhibited Acidobacteria_6 was universal in soil, yet our knowledge of the role of these diverse organisms remained rudimentary^39,40^. Some Actinobacteria are pathogenic to plants. The vast majority of Actinobacteria are important saprophytes capable of decomposing plant and animal debris^41^. Species of Pseudonocardiaceae are recognized as emerging opportunistic pathogens of plants and animals^42^. *Rubrobacter* spp. are extremophiles with radioresistant characteristics^43,44^. *Leucobacter* spp. are generally identified as chromium reducers^45,46^. Certain subspecies of *Leucobacter* have the potential for pathogenic interactions with nematodes^47^. Chloroflexi usually exist in some extreme environments^48,49^. Planctomycetes, Verrucomicrobia, and BRC1 belong to the PVC superphylum. This group appeared to be ubiquitous and contained pathogenic species^50^. *Prosthecobacter* has been found in freshwater, activated sludges, and *Panax notoginseng*^51,52^. Gemmataceae and its genus *Gemmata* have been found in wastewater plants, acid bogs, swamps, and the soil^53,54^. The inhibited taxa were not common disease bacteria. They may be potentially harmful bacteria or useless bacteria on cucumbers. The enriched taxa contained GN02_GKS2_174 and Nannocystaceae. At present, little is known about the ecological function of GN02. Nannocystaceae was reported to promote *Brassica napus* growth^55^.

In addition, network analysis was used to understand how *B. amyloliquefaciens* FH-1 affected the interactions of bacterial communities and to explore whether *B. amyloliquefaciens* FH-1 interacted with the enriched and inhibited taxa. The relative abundance of *B. amyloliquefaciens* was too low; so, a co-occurrence network at the genus level was constructed. The results showed that FH increased the complexity of the whole network, especially the negative interactions. The higher complexity of networks was more resilient to environmental stressors, as different species can complement each other^56^. A negative correlation may mean competition^57^. It is possible that FH increased the competition among bacterial communities and led to the decrease of some taxa and the α-diversity. FH also increased the interactions of *Bacillus* with other genera, especially negative interactions. Except for MB-A2-108 and *Leucobacter* in Actinobacteria, *Bacillus* had no interaction with other inhibited or enriched taxa. This might indicate that *B. amyloliquefaciens* FH-1 does not directly affect the enriched and inhibited taxa. In CK and FH, *Bacillus* and the taxa it interacted with belonged to a module (Fig. 4). Modules reflect the heterogeneity of habitats, aggregation of closely related species in phylogeny, niche overlap, and co-evolution of species^58^. The genera interacting with *Bacillus* in FH (nine phyla) were completely different from those in CK (four phyla) (Table S2). It was speculated that the function of the module with *Bacillus* as the core changed after being inoculated with *B. amyloliquefaciens* FH-1.

Correlation analysis showed that except for GN02_GKS2_174, all inhibited and enriched taxa had significant correlations with cucumber seedlings’ weight and height/length, except root length. This indicated that *B. amyloliquefaciens* FH-1 might promote cucumber seedling growth by regulating the bacterial community and indirectly enriching Nannocystaceae and inhibiting some taxa from Acidobacteria, Actinobacteria, BRC1, Chloroflexi, Plantctomycetes, and Verrucomicrobia. This result was roughly supported by many previously published studies^18,59^. Regulating the rhizosphere microbiome is an important mechanism for PGPB to promote plant growth. Whole genome data showed that *B. amyloliquefaciens* FH-1 had no complete pathway for nitrogen fixation or secretion of IAA, gibberellin (GA), abscisic acid (ABA), or ethylene but had a complete pathway to secrete organic acids (malic acid, acetic acid, succinic acid, and gluconic acid), phytase, zeatin, and siderophore (data not shown). This study showed that FH had no significant effect on soil properties, suggesting that the ability of *B. amyloliquefaciens* FH-1 to dissolve phosphorus and potassium did not play a role in soil characteristics. In our next work, we will verify whether FH promotes cucumber seedling growth by secreting zeatin and siderophore.

## Materials and methods

### Bacterial inoculum preparation

*B. amyloliquefaciens* FH-1 was grown at 30 °C for 48 h in Luria–Bertani (LB) broth on a rotary shaker (180 rpm). The cells were harvested by centrifugation (5000 × *g* for 10 min), and the bacterial pellet was washed three times with 0.9% NaCl and finally resuspended in sterile deionized water at 1 × 10^8^ CFU/ml.

### Pot experiment for the cultivation of cucumber seedlings

For future applications in coastal saline-alkali land, soil (pH 8.14, 4.1 g/kg organic matter, 655 mg/kg total N, 18 mg/kg available N, 250 mg/kg total P, 155 mg/kg available P, 4893 mg/kg total K, and 124 mg/kg available K) was collected from the upper 30 cm of a weed field in an airport economic area in Tianjin, China. The sampled soil was air dried and mixed thoroughly, followed by a sieving step (0.5-cm mesh) to remove plant debris. Cucumber seeds (Jin you NO.1, Tianjin Kerun Agricultural Science Technologies Inc., Tianjin, China) were procured from the local market (Fig. S2). Two cucumber seeds were sown in each plastic pot (diameter 8 cm; height 10 cm) containing 300 g of soil. Pot soils were drenched with 300 ml of the prepared inoculums or equivalent sterile deionized water. In total, there were two treatments: (1) soil drenched with *B. amyloliquefaciens* FH-1 (FH), and (2) soil drenched with sterile deionized water (CK). Five replications of each treatment were set up during the entire experimental period. Pots were placed randomly in a growth chamber at 28 °C day/17 °C night, 75% relative humidity, and 9 h light, and watered weekly. All methods were carried out in accordance with relevant guidelines and regulations.

### Plant characteristics and soil chemical properties

At 35 days after sowing, plants of each pot were harvested and carefully separated into roots and shoots to determine the growth parameters, including length, fresh weight, and dry weight, using rulers and balances. Meanwhile, rhizosphere soil was shaken off of the roots of each treatment to be collected and stored at 4 °C and − 80 °C, respectively.

The rhizosphere soil pH, total organic carbon, total nitrogen, total phosphorus, nitrate nitrogen, and available phosphorus were determined using commercial chemical assay kits (Suzhou Comin Biotechnology Co. Ltd., Suzhou, China) following the manufacturer’s instructions.

### DNA extraction, PCR amplification, and Hiseq sequencing

Soil metagenomic DNA was isolated from 10 soil samples by the PowerSoil DNA isolation kit (MO BIO Laboratories Inc., Carlsbad, CA, USA) according to the manufacturer’s instructions. DNA purity and concentration were monitored by 1% agarose gels and NanoDrop ND-2000 spectrophotometry (NanoDrop Technologies, Wilmington, DE, USA), respectively. The bacterial hypervariable regions (V4) of the 16S rRNA genes were amplified using primer 515F-806R with a barcode^60^. PCR products were purified and sequenced using the Miseq platform at Novogene Co. Ltd (Tianjin, China). The raw sequence data were deposited in the NCBI Sequence Read Archive as accession PRJNA544608 for bacteria. Raw data were processed and analyzed as previously described using the QIIME^60^. The relative abundance of *B. amyloliquefaciens* was determined by local BLAST.

### Data analyses

All statistical analyses were performed using R (version 3.1.1)^61^. The cucumber seedlings’ characteristics, soil properties, bacterial α-diversity indices, and relative abundance of taxa in different treatments were compared using Independent Sample t tests. Principal coordinate analysis (PCoA), analysis of similarity (ANOSIM), and permutational multivariate analysis of variance (PERMANOVA) with the ADONIS function based on weighted UniFrac distance were performed to evaluate the overall differences in the bacterial community^62^. Linear discriminant analysis (LDA) effect size (LEfSe) analysis was used to identify taxa that were enriched and inhibited by FH^63^. Network analysis was used to explore whether FH affected bacteria-bacteria interactions and whether FH directly interacted with the enriched or inhibited taxa at the genus level. The co-occurrence network was inferred based on the Spearman correlation matrix constructed with the 'Hmisc' and 'igraph' package in R. We generated network images and calculated network properties with Gephi^64,65^. Spearman's rank correlation coefficient was used to evaluate the relationships between cucumber seedlings and the taxa enriched and inhibited by FH. Heatmaps that illustrate correlation data were generated using the 'pheatmap' package in R.

## Supplementary Information

Supplementary Information.

## References

[1] Sun Y, Hu KL, Zhang KF, Jiang LH, Xu Y (2012). Simulation of nitrogen fate for greenhouse cucumber grown under different water and fertilizer management using the EU-Rotate_N model. Agric. Water Manage..

[2] Vejan, P., Abdullah, R., Khadiran, T., Ismail, S. & Boyce, A. N. Role of plant growth promoting rhizobacteria in agricultural sustainability—A review. *Molecules* **21**(5), 573. 10.3390/molecules21050573 (2016). 10.3390/molecules21050573 PMC6273255 27136521

[3] Ferreira CMH, Soares H, Soares EV (2019). Promising bacterial genera for agricultural practices: An insight on plant growth-promoting properties and microbial safety aspects. Sci. Total Environ..

[4] Sammauria, R., Kumawat, S., Kumawat, P., Singh, J. & Jatwa, T. K. Microbial inoculants: potential tool for sustainability of agricultural production systems. *Arch. Microbiol.* **202**(4), 677–693 10.1007/s00203-019-01795-w (2020). 10.1007/s00203-019-01795-w 31897539

[5] Singh M (2019). PGPR Amelioration in Sustainable Agriculture.

[6] Berg G (2009). Plant-microbe interactions promoting plant growth and health: perspectives for controlled use of microorganisms in agriculture. Appl. Microbiol. Biotechnol..

[7] Olanrewaju, O. S., Glick, B. R. & Babalola, O. O. Mechanisms of action of plant growth promoting bacteria. *World J. Microbiol. Biotechnol.* **33**(11), 197. 10.1007/s11274-017-2364-9 (2017). 10.1007/s11274-017-2364-9 PMC5686270 28986676

[8] Ambrosini A, de Souza R, Passaglia LMP (2016). Ecological role of bacterial inoculants and their potential impact on soil microbial diversity. Plant Soil.

[9] O'Callaghan M (2016). Microbial inoculation of seed for improved crop performance: Issues and opportunities. Appl. Microbiol. Biotechnol..

[10] Kaminsky LM, Trexler RV, Malik RJ, Hockett KL, Bell TH (2019). The inherent conflicts in developing soil microbial inoculants. Trends Biotechnol..

[11] Chen XH (2007). Comparative analysis of the complete genome sequence of the plant growth-promoting bacterium Bacillus amyloliquefaciens FZB42. Nat. Biotechnol..

[12] Chowdhury, S. P., Hartmann, A., Gao, X. W. & Borriss, R. Biocontrol mechanism by root-associated *Bacillus amyloliquefaciens* FZB42—A review. *Front. Microbiol.* **6**, 780. 10.3389/fmicb.2015.00780 (2015). 10.3389/fmicb.2015.00780 PMC4517070 26284057

[13] Han L (2019). Bacillus amyloliquefaciens B1408 suppresses Fusarium wilt in cucumber by regulating the rhizosphere microbial community. Appl. Soil Ecol..

[14] Wu B (2016). Effects of Bacillus amyloliquefaciens ZM9 on bacterial wilt and rhizosphere microbial communities of tobacco. Appl. Soil Ecol..

[15] Krober, M. *et al.* Effect of the strain *Bacillus amyloliquefaciens* FZB42 on the microbial community in the rhizosphere of lettuce under field conditions analyzed by whole rnetagenome sequencing. *Front. Microbiol.* **5**, 252. 10.3389/fmicb.2014.00252 (2014). 10.3389/fmicb.2014.00252 PMC4033844 24904564

[16] Shen ZZ (2015). Effect of the combination of bio-organic fertiliser with Bacillus amyloliquefaciens NJN-6 on the control of banana Fusarium wilt disease, crop production and banana rhizosphere culturable microflora. Biocontrol Sci. Technol..

[17] Shen ZZ (2015). Rhizosphere microbial community manipulated by 2 years of consecutive biofertilizer application associated with banana Fusarium wilt disease suppression. Biol. Fertility Soils.

[18] Li Q (2019). Rhizosphere microbiome mediated growth-promoting mechanisms of Bacillus amyloliquefaciens FH-1 on rice. Acta Microbiol. Sin..

[19] Qin, Y. X., Shang, Q. M., Zhang, Y., Li, P. L. & Chai, Y. R. *Bacillus amyloliquefaciens* L-S60 reforms the rhizosphere bacterial community and improves growth conditions in cucumber plug seedling. *Front. Microbiol.* **8**, 2620. 10.3389/fmicb.2017.02620 (2017). 10.3389/fmicb.2017.02620 PMC5744474 29312278

[20] Idris EE, Iglesias DJ, Talon M, Borriss R (2007). Tryptophan-dependent production of indole-3-acetic acid (IAA) affects level of plant growth promotion by Bacillus amyloliquefaciens FZB42. Mol. Plant-Microbe Interact..

[21] Mendes R (2011). Deciphering the rhizosphere microbiome for disease-suppressive bacteria. Science.

[22] Panke-Buisse K, Poole AC, Goodrich JK, Ley RE, Kao-Kniffin J (2015). Selection on soil microbiomes reveals reproducible impacts on plant function. ISME J.

[23] de Vries FT, Griffiths RI, Knight CG, Nicolitch O, Williams A (2020). Harnessing rhizosphere microbiomes for drought-resilient crop production. Science.

[24] Rodriguez PA (2019). Systems biology of plant–microbiome interactions. Mol. Plant.

[25] Trabelsi D, Mhamdi R (2013). Microbial inoculants and their impact on soil microbial communities: A review. Biomed. Res. Int..

[26] Gu Y (2020). The effect of microbial inoculant origin on the rhizosphere bacterial community composition and plant growth-promotion. Plant Soil.

[27] Ke XB (2019). Effect of inoculation with nitrogen-fixing bacterium *Pseudomonas stutzeri* A1501 on maize plant growth and the microbiome indigenous to the rhizosphere. Syst. Appl. Microbiol..

[28] Barberan A, Bates ST, Casamayor EO, Fierer N (2012). Using network analysis to explore co-occurrence patterns in soil microbial communities. ISME J..

[29] Kong, Z. Y. *et al.* Co-occurrence patterns of microbial communities affected by inoculants of plant growth-promoting bacteria during phytoremediation of heavy metal contaminated soils. *Ecotoxicol. Environ. Saf.* **183**, 109504. 10.1016/j.ecoenv.2019.109504 (2019). 10.1016/j.ecoenv.2019.109504 31421537

[30] Newman ME (2006). Modularity and community structure in networks. Proc. Natl. Acad. Sci. USA.

[31] Mendes R, Raaijmakers JM (2015). Cross-kingdom similarities in microbiome functions. ISME J..

[32] Mueller UG, Sachs JL (2015). Engineering microbiomes to improve plant and animal health. Trends Microbiol..

[33] Toju H (2018). Core microbiomes for sustainable agroecosystems. Nat. Plants.

[34] Chowdhury, S. P. *et al.* Effects of *Bacillus amyloliquefaciens* FZB42 on lettuce growth and health under pathogen pressure and its impact on the rhizosphere bacterial community. *Plos One* **8**(7), e68818. 10.1371/journal.pone.0068818 (2013). 10.1371/journal.pone.0068818 PMC3720850 23935892

[35] Correa OS (2009). Bacillus amyloliquefaciens BNM122, a potential microbial biocontrol agent applied on soybean seeds, causes a minor impact on rhizosphere and soil microbial communities. Appl. Soil Ecol..

[36] Wan TT, Zhao HH, Wang W (2017). Effect of biocontrol agent Bacillus amyloliquefaciens SN16-1 and plant pathogen Fusarium oxysporum on tomato rhizosphere bacterial community composition. Biol. Control.

[37] Wan TT, Zhao HH, Wang W (2018). Effects of the biocontrol agent Bacillus amyloliquefaciens SN16-1 on the rhizosphere bacterial community and growth of tomato. J. Phytopathol..

[38] Nautiyal CS (2013). Plant growth-promoting bacteria Bacillus amyloliquefaciens NBRISN13 modulates gene expression profile of leaf and rhizosphere community in rice during salt stress. Plant Physiol. Biochem..

[39] Kumar, S., Suyal, D. C., Yadav, A., Shouche, Y. & Goel, R. Microbial diversity and soil physiochemical characteristic of higher altitude. *Plos One* **14**(3), e0213844. 10.1371/journal.pone.0213844 (2019). 10.1371/journal.pone.0213844 PMC6419999 30875404

[40] Kielak, A. M., Barreto, C. C., Kowalchuk, G. A., van Veen, J. A. & Kuramae, E. E. The ecology of acidobacteria: Moving beyond genes and genomes. *Front. Microbiol.* **7**, 744. 10.3389/fmicb.2016.00744 (2016). 10.3389/fmicb.2016.00744 PMC4885859 27303369

[41] Ul-Hassan, A. & Wellington, E. M. Actinobacteria in *Encyclopedia of Microbiology (Third Edition)* (ed Schaechter, M.) 25–44 (Academic Press, 2009).

[42] Zhang M, Powell CA, Guo Y, Benyon L, Duan Y (2013). Characterization of the microbial community structure in Candidatus *Liberibacter asiaticus*-infected citrus plants treated with antibiotics in the field. BMC Microbiol..

[43] Albuquerque L, Johnson MM, Schumann P, Rainey FA, da Costa MS (2014). Description of two new thermophilic species of the genus Rubrobacter, *Rubrobacter calidifluminis* sp. nov. and *Rubrobacter naiadicus* sp. Nov., and emended description of the genus Rubrobacter and the species Rubrobacter bracarensis. Syst. Appl. Microbiol..

[44] Egas, C. *et al.* Complete genome sequence of the radiation-resistant bacterium *Rubrobacter radiotolerans* RSPS-4. *Stand. Genomic. Sci.* **9**(3), 1062–1075. 10.4056/sigs.5661021 (2014). 10.4056/sigs.5661021 PMC4148983 25197483

[45] Ge SM, Zhou MH, Dong XJ, Lu Y, Ge SC (2013). Distinct and effective biotransformation of hexavalent chromium by a novel isolate under aerobic growth followed by facultative anaerobic incubation. Appl. Microbiol. Biotechnol..

[46] Sturm G, Jacobs J, Sproer C, Schumann P, Gescher J (2011). *Leucobacter chromiiresistens* sp. nov., a chromate-resistant strain. Int. J. Syst. Evol. Microbiol..

[47] Muir RE, Tan MW (2008). Virulence of *Leucobacter chromiireducens* subsp. solipictus to *Caenorhabditis elegans*: Characterization of a novel host-pathogen interaction. Appl. Environ. Microbiol..

[48] Zhang Y (2012). Abundance and diversity of candidate division JS1-and Chloroflexi-related bacteria in cold seep sediments of the northern South China Sea. Front. Earth Sci. Prc..

[49] Bennett AC, Murugapiran SK, Hamilton TL (2020). Temperature impacts community structure and function of phototrophic Chloroflexi and Cyanobacteria in two alkaline hot springs in Yellowstone National Park. Environ. Microbiol. Rep..

[50] Devos DP (2013). Gemmata obscuriglobus. Curr. Biol..

[51] Dong, L. L. *et al.* Diversity and composition of bacterial endophytes among plant parts of *Panax notoginseng*. *Chin. Med. UK* **13**, 41. 10.1186/s13020-018-0198-5 (2018). 10.1186/s13020-018-0198-5 PMC6092820 30127840

[52] Ma Q (2015). Bacterial community compositions of coking wastewater treatment plants in steel industry revealed by Illumina high-throughput sequencing. Bioresour. Technol..

[53] Kepel BJF, Gani MA, Tallei TE (2020). Comparison of bacterial community structure and diversity in traditional gold mining waste disposal site and rice field by using a metabarcoding approach. Int. J. Microbiol..

[54] Chouari R (2003). Molecular evidence for novel planctomycete diversity in a municipal wastewater treatment plant. Appl. Environ. Microbiol..

[55] Zhao, Y. *et al.* Endosphere microbiome comparison between symptomatic and asymptomatic roots of *Brassica napus* infected with *Plasmodiophora brassicae*. *Plos One* **12**(10), e0185907. 10.1371/journal.pone.0185907 (2017). 10.1371/journal.pone.0185907 PMC5655474 29065162

[56] Banerjee S (2019). Agricultural intensification reduces microbial network complexity and the abundance of keystone taxa in roots. ISME J..

[57] Faust K, Raes J (2012). Microbial interactions: From networks to models. Nat. Rev. Microbiol..

[58] Olesen JM, Bascompte J, Dupont YL, Jordano P (2007). The modularity of pollination networks. Proc. Natl. Acad. Sci. USA.

[59] Kang Y, Shen M, Wang H, Zhao Q (2013). A possible mechanism of action of plant growth-promoting rhizobacteria (PGPR) strain *Bacillus pumilus* WP8 via regulation of soil bacterial community structure. J. Gen. Appl. Microbiol..

[60] Wang J (2018). Traits-based integration of multi-species inoculants facilitates shifts of indigenous soil bacterial community. Front. Microbiol..

[61] R Core Team. R: A language and environment for statistical computing. *R Foundation for Statistical Computing* (2020).

[62] Xiong J (2015). Evidence of bacterioplankton community adaptation in response to long-term mariculture disturbance. Scientific Report.

[63] Segata, N. *et al.* Metagenomic biomarker discovery and explanation. *Genome Biol.* **12**(6), R60. 10.1186/gb-2011-12-6-r60 (2011). 10.1186/gb-2011-12-6-r60 PMC3218848 21702898

[64] Jiang YJ (2017). Plant cultivars imprint the rhizosphere bacterial community composition and association networks. Soil Biol. Biochem..

[65] Ju F, Xia Y, Guo F, Wang ZP, Zhang T (2014). Taxonomic relatedness shapes bacterial assembly in activated sludge of globally distributed wastewater treatment plants. Environ. Microbiol..

