# Supplementary material for: Bacillus amyloliquefaciens FH-1 significantly affects cucumber seedlings and the rhizosphere bacterial community but not soil
Source: Sci Rep. 2021 Jun 8;11:12055. doi: 10.1038/s41598-021-91399-6 (PMC8187646; doi:10.1038/s41598-021-91399-6)
Supplement: Supplementary file 1 — Supplementary Information. [file 41598_2021_91399_MOESM1_ESM.docx]

***Bacillus amyloliquefaciens* FH-1 significantly affected cucumber seedlings and rhizosphere bacterial community but not soil**

Jingjing Wang^ab*^, Song Xu^ab^, Rong Yang^ab^, Wei Zhao^ab^, Dan Zhu^ab^, Xiaoxia Zhang^ab^, Zhiyong Huang^ab*^

^a^Tianjin Key Laboratory for Industrial Biological Systems and Bioprocessing Engineering, Tianjin Institute of Industrial Biotechnology, Chinese Academy of Sciences, Tianjin 300308, China

^b^National Technology Innovation Center of Synthetic Biology, Tianjin 300308, China

^*^ **Corresponding author:** Jingjing Wang

Tianjin Institute of Industrial Biotechnology, Chinese Academy of Sciences

No. 32, West 7th Road

Tianjin Airport Economic Area

Tianjin 300308, P R China

Tel.: +86-22-84861930; Fax: +86-22-84861930

E-mail address: [wang_jj@tib.cas.cn](mailto:huang_zy@tib.cas.cn)

**A B**

**Fig. S1.** Relative abundances of *Bacillus amyloliquefaciens* (A) and *Bacillus* (B) under different treatments. CK, non-inoculated; FH, inoculated with *Bacillus amyloliquefaciens* FH-1.


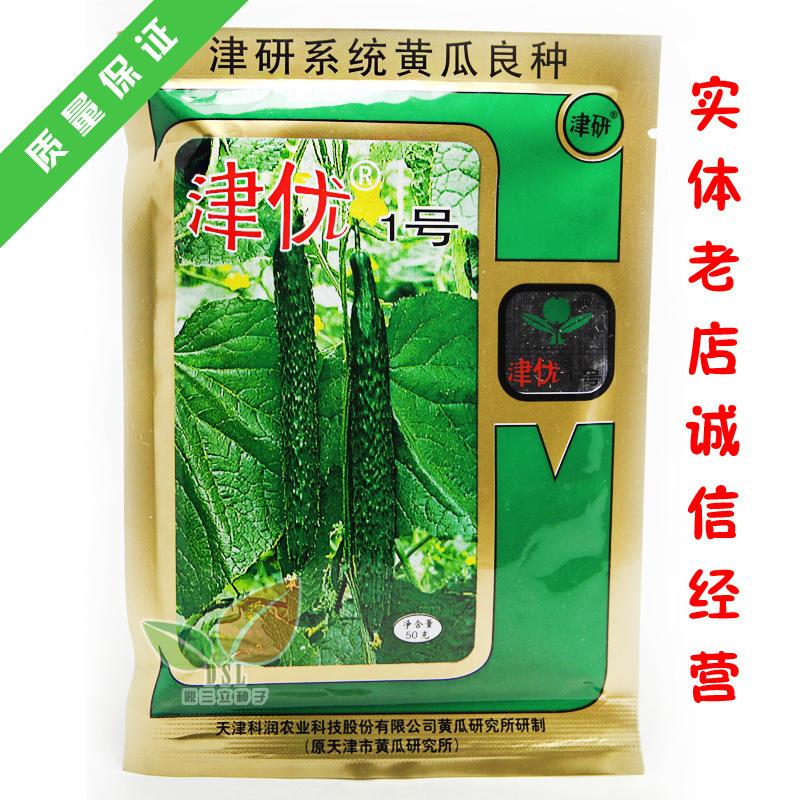

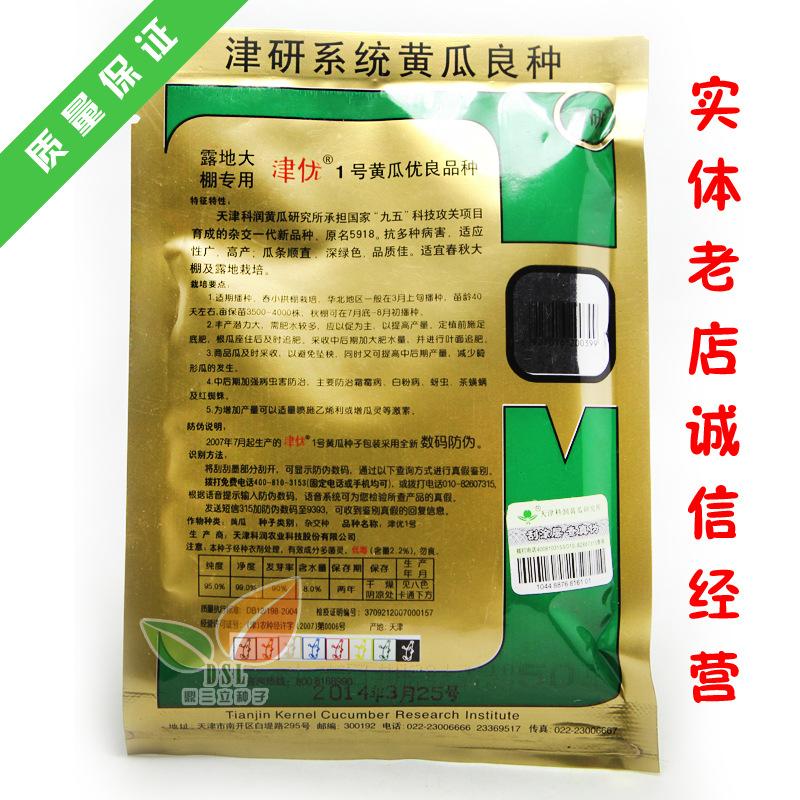


**Fig. S2.** Cucumber seeds used in this manuscript

**Table S1** Effects of *Bacillus amyloliquefaciens* FH-1 inoculations on relative abundance of bacterial phylum (proteobacterial classes)

|  | **CK** | **FH** |
| --- | --- | --- |
| Alphaproteobacteria | 18.18 ± 1.77a | 19.37 ± 1.49a |
| Actinobacteria | 16.43 ± 1.95a | 14.73 ± 1.38a |
| **Acidobacteria** | **11.03 ± 1.00a** | **8.06 ± 1.18b** |
| Betaproteobacteria | 11.10 ± 1.85a | 13.37 ± 1.32a |
| Gammaproteobacteria | 8.39 ± 1.59a | 8.15 ± 0.79a |
| **Deltaproteobacteria** | **6.64 ± 0.32b** | **7.51 ± 0.47a** |
| Gemmatimonadetes | 7.25 ± 0.97a | 7.61 ± 0.53a |
| Bacteroidetes | 6.75 ± 0.72a | 8.87 ± 2.48a |
| Chloroflexi | 4.05 ± 0.55a | 3.44 ± 0.58a |
| Planctomycetes | 2.60 ± 0.74a | 1.67 ± 0.75a |
| Firmicutes | 2.37 ± 0.57a | 2.28 ±0.23a |
| Verrucomicrobia | 2.22 ± 0.12a | 1.96 ± 0.65a |
| Nitrospirae | 0.75 ± 0.12a | 0.80 ± 0.15a |
| Armatimonadetes | 0.60 ± 0.02a | 0.58± 0.07a |
| Cyanobacteria | 0.52 ± 0.05a | 0.60 ± 0.15a |
| TM7 | 0.22 ± 0.03a | 0.22 ± 0.06a |
| Fibrobacteres | 0.18 ± 0.05a | 0.15 ± 0.03a |
| Chlorobi | 0.19± 0.06a | 0.18 ± 0.03a |
| Others | 0.46 ± 0.04a | 0.39 ± 0.05b |

CK, non-inoculated; FH, inoculated with *Bacillus amyloliquefaciens* FH-1. Values (means ± SD, n=5) within the same row followed by different letters are significantly different at *P* < 0.05 according to Independent-Samples t Test.

**Table S2** Interactions of *Bacillus* in different treatments

| **Source** | **Target** | **Source/Targe Affiliations** | **Correlations**  **(*P*<0.05)** | |
| --- | --- | --- | --- | --- |
|  |  |  | **CK** | **FH** |
| **n65** | **n65** | Firmicutes;c__Bacilli;o__Bacillales;f__Bacillaceae;g__Bacillus |  |  |
|  |  |  |  |  |
| **n30** | n65 | Proteobacteria;c__Alphaproteobacteria;o__Rhizobiales;f__Bradyrhizobiaceae;g__Balneimonas | 1 |  |
| **n36** | n65 | Proteobacteria;c__Deltaproteobacteria;o__Myxococcales;f__Haliangiaceae;g__ | -1 |  |
| n65 | **n160** | Proteobacteria;c__Alphaproteobacteria;o__Rhizobiales;f__Hyphomicrobiaceae;g__Pedomicrobium | 1 |  |
| n65 | **n337** | Bacteroidetes;c__Cytophagia;o__Cytophagales;f__;g__ | 1 |  |
| n65 | **n365** | Firmicutes;c__Bacilli;o__Bacillales;f__Paenibacillaceae;g__Cohnella | 1 |  |
| n65 | **n478** | Proteobacteria;c__Alphaproteobacteria;o__Rickettsiales;f__Rickettsiaceae;g__Rickettsia | 1 |  |
| n65 | **n532** | Actinobacteria;c__Actinobacteria;o__Actinomycetales;f__Microbacteriaceae;g__Leucobacter | 1 |  |
|  |  |  |  |  |
| **n4** | n65 | Bacteroidetes;c__Cytophagia;o__Cytophagales;f__Cytophagaceae;g__Pontibacter |  | 1 |
| **n15** | n65 | Gemmatimonadetes;c__Gemm-5;o__;f__;g__ |  | -1 |
| **n23** | n65 | Gemmatimonadetes;c__Gemm-3;o__;f__;g__ |  | -1 |
| **n24** | n65 | Proteobacteria;c__Alphaproteobacteria;o__Rhodospirillales;f__;g__ |  | 1 |
| **n25** | n65 | Firmicutes;c__Bacilli;o__Bacillales;Other;Other |  | 1 |
| **n26** | n65 | Verrucomicrobia;c__[Pedosphaerae];o__[Pedosphaerales];f__Ellin517;g__ |  | 1 |
| **n52** | n65 | Actinobacteria;c__Thermoleophilia;o__Solirubrobacterales;f__Solirubrobacteraceae;g__ |  | -1 |
| n65 | **n102** | Armatimonadetes;c__[Fimbriimonadia];o__[Fimbriimonadales];f__[Fimbriimonadaceae];g__Fimbriimonas |  | 1 |
| n65 | **n134** | Actinobacteria;c__Actinobacteria;o__Actinomycetales;f__Micromonosporaceae;g__ |  | -1 |
| n65 | **n135** | Acidobacteria;c__Acidobacteria-5;o__;f__;g__ |  | 1 |
| n65 | **n165** | Actinobacteria;c__Actinobacteria;o__Actinomycetales;f__Geodermatophilaceae;g__Geodermatophilus |  | -1 |
| n65 | **n181** | Chloroflexi;c__Thermomicrobia;o__AKYG1722;f__;g__ |  | -1 |
| n65 | **n200** | Actinobacteria;c__MB-A2-108;o__;f__;g__ |  | 1 |
| n65 | **n379** | Proteobacteria;c__Gammaproteobacteria;o__34P16;f__;g__ |  | -1 |
| n65 | **n409** | Chloroflexi;c__SHA-26;o__;f__;g__ |  | -1 |
| n65 | **n467** | Chloroflexi;c__Chloroflexi;o__Chloroflexales;f__Oscillochloridaceae;g__Oscillochloris |  | -1 |
| n65 | **n515** | Proteobacteria;c__Alphaproteobacteria;o__Rhizobiales;f__Methylobacteriaceae;g__ |  | -1 |
| n65 | **n594** | Proteobacteria;c__Deltaproteobacteria;o__Desulfuromonadales;Other;Other |  | -1 |
| n65 | **n606** | Proteobacteria;c__Gammaproteobacteria;o__Xanthomonadales;f__Xanthomonadaceae;Other |  | -1 |

CK, non-inoculated; FH, inoculated with *Bacillus amyloliquefaciens* FH-1.

**Table S3** The effects of *Bacillus amyloliquefaciens* inoculation on bacterial community

| **Strains** | **Plants** | **Experiments** | **Types** | **Methods** | **Impacts on bacterial community** | | **References** |
| --- | --- | --- | --- | --- | --- | --- | --- |
|  |  |  |  |  | **α-Diversity** | **Composition** |  |
| FH1 | Rice | Field | Biofertilizer | Miseq (V4-V5) | Significantly decreased | Significantly increased γ-Proteobacteria and Chloroflexi while decreased β-Proteobacteria and Actinobacteria | [1] |
| B1408 | Cucumber | Pot  (peat and vermiculite) | Biocontrol | Miseq (V4-V5) | Significantly increased | Relatively increased Firmicutes and Chloroflexi | [2] |
| SN16-1 | Tomato | Pot  (soil and perlite) | Biocontrol | T-RFLP | Transient influence | | [3] |
| DT | -- | Bottle  (soil) | Ammonifier | HiSeq (V3-V4) | Significantly decreased | Firmicutes and Actinobacteria were enriched | [4] |
| SN16-1 | Tomato | Pot  (soil and perlite) | Biocontrol | MiSeq (V4) | Transient effects | | [5] |
| L-S60 | Cucumber | Plug tray  (peat moss, vermiculite, and perlite) | Biofertilizer | HiSeq (V3-V4) | Slightly lowered | Decreased Acidobacteria and Gemmatimonadetes while increased Firmicutes | [6] |
| PTS-394 | Tomato | Pot  (vermiculite, rice field soil and organic manure) | Biofertilizer | 454 (V1-V3) | No durable impact | | [7] |
| ZM9 | Tobacco | Field | Biocontrol | Hiseq (V4) | Increased except LSFC in 1 and 5 week | Significantly increased Proteobacteria while decreased Acidobacteria one week after irrigation | [8] |
| NJN6 | Banana | Field | Biocontrol | 454 (V4-V5) | Significantly increased | The abundance of Acidobacteria (Gp1 and Gp3) and Firmicutes, was significantly increased, while the abundance of Proteobacteria was significantly decreased | [9] |
| NJN6 | Banana | Field | Biocontrol | DGGE (V3) | Significantly increased | Enriched the genera Comamonas, Chitinophaga, the species Bacillus flexus and uncultured Bacillus | [10] |
| FZB42 | Lettuce | Field | Biofertilizer | Miseq  (Whole metagenome) | Marginal changes | | [11] |
| FZB42 | Lettuce | Tray  (quartz sand and substrate) | Biocontrol | 454 (Gammaproteobacteria) | Decreased diversity causing by pathogen |  | [12] |
| NBRISN13 | Rice | Pot  (soil) | Salt-resistant | Biolog | Lowered but increased under salt | Stimulated population of betaine-, sucrose-, trehalose-, and glutamine-utilizing bacteria in salt-treated rice rhizosphere | [13] |
| FZB42 | Lettuce | Field | Biocontrol | T-RFLP (27F-907R) | No durable impact | | [14] |
| BNM122 | Soybean | Pot  (soil) | Biocontrol | DGGE / Biolog | Minor impact | | [15] |

**REFERENCES**

1. Li, Q., et al., *Rhizosphere microbiome mediated growth-promoting mechanisms of Bacillus amyloliquefaciens FH-1 on rice.* Acta Microbiologica Sinica, 2019. **59**(10): p. 1-17.

2. Han, L., et al., *Bacillus amyloliquefaciens B1408 suppresses Fusarium wilt in cucumber by regulating the rhizosphere microbial community.* Applied Soil Ecology, 2019. **136**: p. 55-66.

3. Wan, T.T., H.H. Zhao, and W. Wang, *Effects of the biocontrol agent Bacillus amyloliquefaciens SN16-1 on the rhizosphere bacterial community and growth of tomato.* Journal of Phytopathology, 2018. **166**(5): p. 324-332.

4. Hui, C., et al., *Shifts in microbial community structure and soil nitrogen mineralization following short-term soil amendment with the ammonifier Bacillus amyloliquefaciens DT.* International Biodeterioration & Biodegradation, 2018. **132**: p. 40-48.

5. Wan, T.T., H.H. Zhao, and W. Wang, *Effect of biocontrol agent Bacillus amyloliquefaciens SN16-1 and plant pathogen Fusarium oxysporum on tomato rhizosphere bacterial community composition.* Biological Control, 2017. **112**: p. 1-9.

6. Qin, Y.X., et al., *Bacillus amyloliquefaciens L-S60 Reforms the Rhizosphere Bacterial Community and Improves Growth Conditions in Cucumber Plug Seedling.* Frontiers in Microbiology, 2017. **8**.

7. Qiao, J.Q., et al., *Addition of plant-growth-promoting Bacillus subtilis PTS-394 on tomato rhizosphere has no durable impact on composition of root microbiome.* Bmc Microbiology, 2017. **17**: p. 1-12.

8. Wu, B., et al., *Effects of Bacillus amyloliquefaciens ZM9 on bacterial wilt and rhizosphere microbial communities of tobacco.* Applied Soil Ecology, 2016. **103**: p. 1-12.

9. Shen, Z.Z., et al., *Rhizosphere microbial community manipulated by 2 years of consecutive biofertilizer application associated with banana Fusarium wilt disease suppression.* Biology and Fertility of Soils, 2015. **51**(5): p. 553-562.

10. Shen, Z.Z., et al., *Effect of the combination of bio-organic fertiliser with Bacillus amyloliquefaciens NJN-6 on the control of banana Fusarium wilt disease, crop production and banana rhizosphere culturable microflora.* Biocontrol Science and Technology, 2015. **25**(6): p. 716-731.

11. Krober, M., et al., *Effect of the strain Bacillus amyloliquefaciens FZB42 on the microbial community in the rhizosphere of lettuce under field conditions analyzed by whole rnetagenome sequencing.* Frontiers in Microbiology, 2014. **5**.

12. Erlacher, A., et al., *The impact of the pathogen Rhizoctonia solani and its beneficial counterpart Bacillus amyloliquefaciens on the indigenous lettuce microbiome.* Frontiers in Microbiology, 2014. **5**.

13. Nautiyal, C.S., et al., *Plant growth-promoting bacteria Bacillus amyloliquefaciens NBRISN13 modulates gene expression profile of leaf and rhizosphere community in rice during salt stress.* Plant Physiology and Biochemistry, 2013. **66**: p. 1-9.

14. Chowdhury, S.P., et al., *Effects of Bacillus amyloliquefaciens FZB42 on Lettuce Growth and Health under Pathogen Pressure and Its Impact on the Rhizosphere Bacterial Community.* Plos One, 2013. **8**(7).

15. Correa, O.S., et al., *Bacillus amyloliquefaciens BNM122, a potential microbial biocontrol agent applied on soybean seeds, causes a minor impact on rhizosphere and soil microbial communities.* Applied Soil Ecology, 2009. **41**(2): p. 185-194.
